# Efficient arabinoxylan assay for wheat: Exploring variability and molecular marker associations in Wholemeal and refined flour

**DOI:** 10.1016/j.jcs.2024.103897

**Published:** 2024-05

**Authors:** Nayelli Hernández-Espinosa, Gabriel Posadas-Romano, Susanne Dreisigacker, Jose Crossa, Leonardo Crespo, Maria Itria Ibba

**Affiliations:** Global Wheat program, International Maize and Wheat Improvement Center (CIMMYT), Km. 45 Carretera México-Veracruz, Texcoco, Edo. de México, CP 56100, México

**Keywords:** High-throughput assay, Arabinoxylan, Dietary fibers, Molecular markers

## Abstract

In this study, we present a modified high throughput phloroglucinol colorimetric assay for the quantification of arabinoxylans (AX) in wheat named PentoQuant. The method was downscaled from a 10 ml glass tube to 2 ml microcentrifuge tube format, resulting in a fivefold increase in throughput while concurrently reducing the overall cost and manual labor required for the analysis. Comparison with established colorimetric assays and gas chromatography validates the modified protocol, demonstrating its superior repeatability, rapidity, and simplicity. The effectiveness of the protocol was tested on 606 unique whole meal (WM) and refined flour (RF) bread wheat samples which revealed the presence of more than a twofold variation in both the soluble (WE-AX) and total (TOT-AX) AX fractions in WM (TOT-AX = 31.9–76.1 mg/g; WE-AX = 4.4–12.6 mg/g) and RF (TOT-AX = 7.7–22.4 mg/g; WE-AX = 3.9–11.4 mg/g). Results obtained from the AX quantification were used to test the effectiveness of four molecular markers associated with AX variation and targeting two major genomic regions on the 1BL and 6BS chromosomes. These markers appeared to be particularly relevant for the WE-AX fraction, providing insights to enable marker-assisted breeding.

## Highlights

• PentoQuant development: a modified colorimetric assay for arabinoxylans quantification.
• PentoQuant improves efficiency, throughput, and repeatability while cutting costs.
• More than two-fold TOT and WE-AX variation identified among 606 flour samples.
• Lines with high WE-AX could be identified using the 1B and 6B QTL KASP markers.

## Graphical abstract

**Figure:**
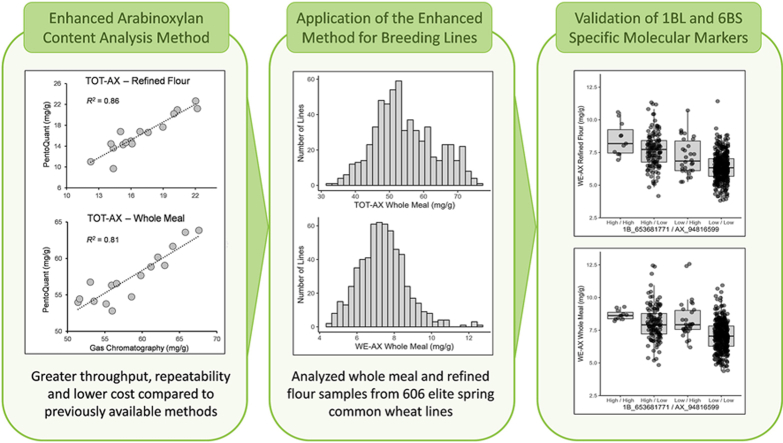

## 1 Introduction

The three staple cereals – wheat, rice, maize – comprise a major component of the human diet, accounting for nearly half of the world's food calories and two-fifths of protein intake (Erenstein et al., 2022). Wheat alone plays a particularly crucial role in ensuring global food and nutrition security, contributing to around 20% of the total dietary calories and proteins worldwide (Shiferaw et al., 2013) and constitutes a major source of other components beneficial for human health such as dietary fibers (DF) (Ibba et al., 2021). Epidemiological studies have shown that diets with high DF and whole grain consumption are associated with diminished risk of coronary heart disease, colon cancers, inflammatory bowel disease, and metabolic syndrome (Stephen et al., 2017). For this reason, health authorities around the world recommend that adults consume 25 g–35 g of fiber per day (Lovegrove et al., 2020; Stephen et al., 2017). However, despite these established health benefits, the intake of DF in most countries falls far below the recommended levels which makes DF a component of public health concern.

The wheat grain contains around 12–14% of DF which are mostly located in the bran. Arabinoxylans (AX) are the major component of DF and constitute around 50% of the wheat TDF (Barron et al., 2020). AX consist of a linear backbone of β-1,4 linked D-xylopyranosyl residues. These residues may be unsubstituted, monosubstituted, or disubstituted with α-L-arabinofuranose residues at the second and/or third carbon position, and the resulting polymer chain adopts a random coil conformation. Some arabinose moieties can be esterified with ferulic acid at the fifth carbon position (Ramseyer et al., 2011). Variations in the length of the xylose backbone, substitution of the xylose units with arabinose, substitution of the arabinose units with ferulic acid, and the overall molecular weight of the AX molecule, greatly affect the functionality, and solubility of AX, which are typically divided into water extractable (WE-AX) and water unextractable (WU-AX) AX (Kiszonas et al., 2013).

Various studies have reported different methods for AX extraction and quantification. The most common methods for quantifying AX involve determining arabinose and xylose through gas or anion-exchange chromatography, or globally measuring pentose with the phloroglucinol colorimetric assay. Both gas and anion-exchange chromatography allow for the quantification of the monosaccharides released after the acid hydrolysis of AX polymers. These two methods are probably the most accurate for the AX quantification and are widely adopted. In both cases however, the sample preparation process is lengthy, the throughput of the method is low, and the analysis requires a high degree of technical skills, expensive instrumentation, and the generation of calibration curves using standard sugars. For these reasons, adoption of chromatography for the routine analysis of AX content cannot be easily implemented within a breeding scheme (Gebruers et al., 2008; Saulnier et al., 1995; Snelders et al., 2013). The phloroglucinol colorimetric assay is based on the acid hydrolysis of the five-carbon monosaccharides that make up the AX polymer, into furfural. The furfural is then allowed to react with phloroglucinol, producing a colored phloroglucide whose intensity depends on the pentosans content (Kiszonas et al., 2012). This method was first developed by Douglas (1981) and since then it has been widely adopted to measure the AX content in wheat flour using different modifications (Finnie et al., 2006; Hernández-Espinosa et al., 2020; Kiszonas et al., 2012). The success of this method is mostly determined by the relatively low cost and accessibility of the assay. Nevertheless, in the current form, this method has low throughput, requires skilled personnel, is associated with a relatively low repeatability, and uses toxic and corrosive chemicals. Modification of this assay to make it more accessible to different laboratories and to be used for the analysis of several genotypes within a breeding program, are desirable.

Despite the different challenges associated with the AX quantification, several authors have investigated the variability of wheat grain AX content and its genetic basis using different extraction and quantification methods (Finnie et al., 2006; Ramseyer et al., 2011; Saulnier et al., 2007). According to the results obtained from different linkage mapping and genome-wide association studies, two major Quantitative Trait Loci (QTL) associated with significant changes in total and water-extractable AX content, have been identified on the long arm of chromosome 1B and the short arm of chromosome 6B, in addition to several minor effect QTL (Charmet et al., 2009; Ibba et al., 2021; Lovegrove et al., 2020; Martinant et al., 1998; Quraishi et al., 2011). Kompetitive Allele Specific PCR (KASP) markers targeting the 1B QTL have been developed by Lovegrove et al. (2020) and Ibba et al. (2021). These markers have been validated in a limited set of samples where they proved to be significantly associated with AX content variation. More studies will be needed to confirm the value of these markers across different germplasm. Recently, a marker specific for the 6B QTL, has also been developed (Mitchell et al., 2023). This marker targets a peroxidase gene that is thought to be responsible for the observed variations in the WE-AX content. Also in this case, the effectiveness of the marker should be tested across different germplasm to confirm its association with AX content.

To develop wheat lines with improved AX is of fundamental importance to understand the variability of AX content and the genomic regions responsible for its synthesis. It is also crucial to have efficient biochemical and molecular tools for the characterization of AX content and the rapid selection of high-AX lines. For these reasons, the objectives of this research were to: a) develop a reliable, affordable and rapid method for AX quantification ideal for screening large numbers of wheat lines in breeding programs, b) evaluate the AX variation of different genotypes for total (TOT-AX) and WE-AX fractions in wheat whole meal and refined flour and c) validate the KASP markers reported by Lovegrove et al. (2020), Ibba et al. (2021) and Mitchell et al. (2023) and evaluate their association with changes in AX concentration in both whole meal and refined flour. The results of this research will contribute to improving the knowledge on wheat AX variability and evaluation methods and will provide useful tools for the improved selection of high AX lines within a wheat breeding program.

## 2 Materials and methods

### 2.1 Plant material

The study was conducted on 606 spring common wheat genotypes developed by the CIMMYT spring bread wheat breeding program. All the lines were grown during the 2017–2018 cropping season at Campo Experimental Norman E. Borlaug (CENEB) in Ciudad Obregón, Sonora (northwest Mexico) under optimal conditions. All genotypes were planted in December 2017 and harvested in May 2018. Plots were managed following standard agronomic practices for the site. The PentoQuant method validation was conducted using 16 spring common wheat genotypes developed by CIMMYT and grown under optimal conditions at the CENEB experimental station during the 2018–2019 cycle.

### 2.2 Milling procedure

Whole meal was obtained using approximately 2 g of grains which were processed using a cyclone mill equipped with a 0.5 mm mesh, obtaining flour with particles of this size and smaller. Immediately after grinding, all the samples were thoroughly homogenized. Refined flour samples were obtained using a Brabender Quadrumat Senior mill (C.W. Brabender OHG, Germany). Before milling, all the samples were tempered according to their hardness and following the official AACC method 26–95.01 (AACC Approved Methods of Analysis, 2011). Grain hardness was measured using the Single Kernel Characterization System (SKCS 4100 Model, Perten) following AACC method 55-31.01 (AACC Approved Methods of Analysis, 2011).

### 2.3 Arabinoxylan (AX) determination using the PentoQuant method

**Supplementary Fig. 1:**
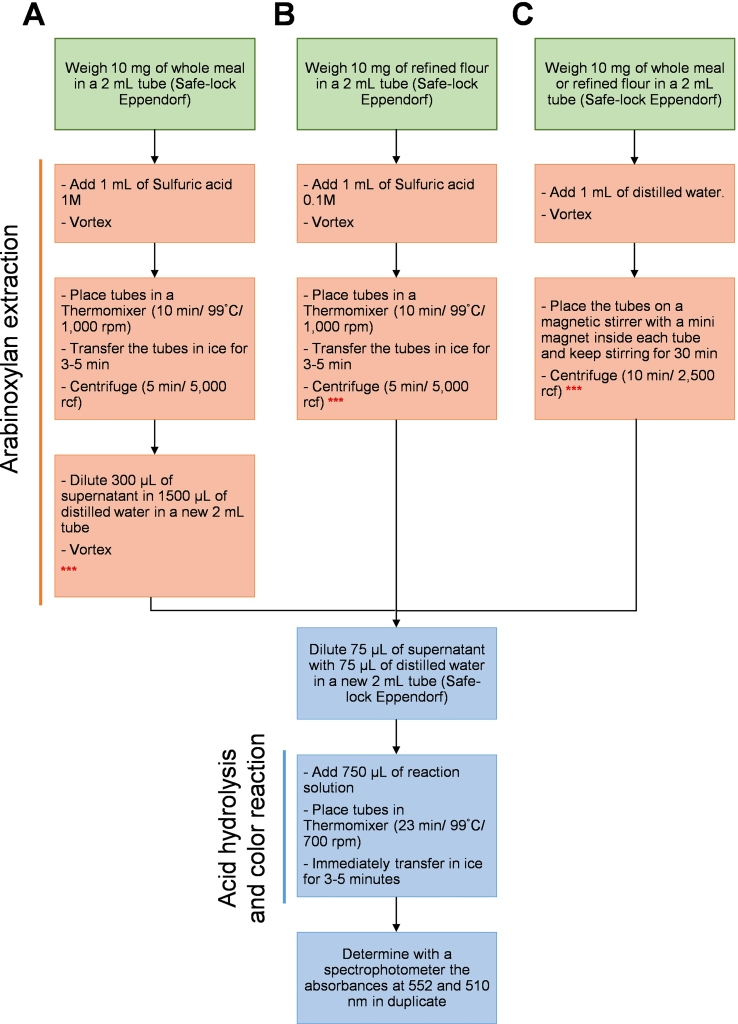
Schematic representation of the protocol used to determine (A) the TOT-AX content in whole meal; (B) the TOT-AX content in refined flour; and (C) the WE-AX content in whole meal and refined flour. During the color reaction, it is important to use the Eppendorf Safe-Lock microcentrifuge tubes to ensure the correct color development. For the extraction of the TOT-AX from whole meal, it is important to use the correct reaction solution which employs a 20% phloroglucinol solution. For the quantification of the TOT-AX in refined flour and the WE-AX in both whole meal and refined flour, the reaction solution employs a 10% phloroglucinol solution. After the AX extraction (***) it is possible to store at −20 °C the samples and continue with the rest of the reaction another day.

The content of AX in this study was determined in duplicate using a modified version of the colorimetric method reported by Hernández-Espinosa et al. (2020) which was developed based on the protocol reported by Finnie et al. (2006) and by Rouau & Surget (1994). This optimized colorimetric method has been named “PentoQuant”. A detailed description of the method is reported in Hernández-Espinosa et al. (2022). A schematic representation of the method is reported in Supplementary Fig. 1. Briefly, 10 mg (±0.5 mg) of whole meal (WM) or refined flour (RF) were weighted in 2 ml microcentrifuge tubes (Eppendorf, Hamburg, Germany) for the extraction of either the WE-AX or the TOT-AX fraction. For WE-AX, the RF or WM sample was suspended in 1 ml distilled water, mixed for 30 min in a platform rocker and centrifuged for 10 min at 2500 rcf. For the TOT-AX, refined flour sample was suspended in 1 ml of 0.1M sulfuric acid, mixed with a vortex, placed in a Thermomixer (Eppendorf, Hamburg, Germany) for 10 min at 1000 rpm and 99 °C, and then centrifuged for 5 min at 5000 rcf. A similar procedure was applied for TOT-AX in whole meal, except that samples were suspended in 1 ml of 1M Sulfuric Acid and after centrifugation, 300 μl of the extracted sample were diluted in 1500 μl of distilled water. Finally, 75 μl of supernatant from either the WE-AX or TOT-AX extraction sample, was transferred in a 2 ml Eppendorf safe-lock microcentrifuge tube together with 75 μl of distilled water and 750 μl of reaction solution. One liter of reaction solution contains 932 ml of acetic acid, 17 ml of concentrated hydrochloric acid, 42.4 ml of phloroglucinol 20% (w/v) in ethanol (10% in refined flour), and 8.5 ml of glucose (1.75% w/v). The microcentrifuge tubes were then placed in a Thermomixer (Eppendorf, Hamburg, Germany) (23 min/700 rpm/99 °C) and later immediately transferred in ice-cold water for about 2 min. Finally, 300 μl of the solutions were placed into a 96-well microplate in duplicate where their absorbance at 552 and 510 nm was measured using an Epoch microplate spectrophotometer (BioTek, Winooski, VT, U.S.A.). The AX content was determined based on a calibration curve generated with known quantities of xylose and using the equations that were presented in previous reports (Hernández-Espinosa et al., 2020, 2022). The average arabinoxylan content values obtained using the PentoQuant method, are available at the following link https://hdl.handle.net/11529/10548997.

### 2.4 Optimization of the PentoQuant phloroglucinol colorimetric assay

Optimization of the PentoQuant method was conducted through the alteration of several parameters which included: 1) changes in the phloroglucinol concentration in ethanol at 5%, 10%, 15%, 20%, 25%, 30% and 35% (w/v); 2) the quantity of flour (10 and 30 mg) used for the extraction; 3) the method of extraction of TOT-AX (with or without sulfuric acid); 4) the whole meal grinding method (Cyclone mill with a 0.5 mm mesh or a ball mill run at 30 Hz/3 min/2 g grain); 5) the reaction time (10, 20 and 30 min); 6) the reaction temperature (90, 95, 97, 98 and 99 °C); 7) the centrifuge speed (6000/5000/3000/2000 rcf) and time (5 or 3 min) after the extraction, and 8) the volumes and ratios of the dilutions (75 μl:75 μl, 300 μl: 300 μl; 300 μl: 900 μl; 150 μl: 1650 μl; 300 μl: 1500 μl). All the above-mentioned alternations were examined either alone or in combination to verify the function of each parameter on the AX quantification.

### 2.5 Gas chromatography

The TOT-AX fraction of 16 different flour samples was analyzed according to the method reported by Saulnier et al. (1995). Specifically, 10 mg of RF and WM, were hydrolyzed in 1M H_2_SO_4_ at 100 °C for 2 h and the released individual neutral sugars were converted into alditol acetate and quantified by gas chromatography (GC). Arabinoxylan content was calculated as the sum of arabinose and xylose.

### 2.6 Molecular markers analysis

The KASP markers used to characterize the wheat lines selected for the study were marker BA00789946 reported by Lovegrove et al. (2020), 1B_653086336 and 1B_653681771 reported by Ibba et al. (2021) and the AX_94816599 reported by Mitchell et al. (2023). Marker analysis was performed using reactions containing 2 μl water, 2 μl 1 × PACE genotyping master mix (https://3crbio.com), 0.07 μl assay mix and 3 μl to 50 ng of dried DNA with a PCR profile of 95 °C for 15 min activation time, followed by 10 cycles of 95 °C for 20 s and 65 °C for 25 s (drop −1 °C each cycle), followed by 30 cycles of 95 °C for 10 s and 57 °C for 60 s. Fluorescence was read as an end point reading at 25 °C. The results obtained from the molecular marker analysis are available at the following link https://hdl.handle.net/11529/10548997.

### 2.7 Statistical analysis

Pearson correlation coefficients (*r*) and statistical analyses were performed using the SAS ® OnDemand for Academics (https://www.sas.com/en_us/software/on-demand-for-academics.html). The analysis of variance and comparison of least square means was performed using the PROC GLM procedure and using the Fisher's protected LSD at the α = 0.05 significance level.

## 3 Results and discussion

### 3.1 Optimization and validation of the PentoQuant phloroglucinol colorimetric assay

We report a modification of the phloroglucinol colorimetric assay originally developed by Douglas (1981), which combines the procedure previously reported by Hernández-Espinosa et al. (2020) and Rouau & Surget (1994). The method was scaled from a 10 ml screw cap Pyrex glass tube to a 2 ml Eppendorf safe-lock tube format which allowed for the analysis of 100 lines per day by a single laboratory technician compared to the 20 lines per day analyzed by two laboratory technicians following the protocol reported by Hernández-Espinosa et al. (2020). This method, here named “PentoQuant”, greatly improves the efficiency, throughput, and repeatability of the original protocol, being ideal for the screening of larger numbers of wheat lines within a breeding program. Several parameters were altered for the method optimization, testing changes that involved the type and quantity of the flour, and key steps and/or solutions used during the AX extraction process and color reaction. Among these factors, the whole meal grinding method was not associated with significant differences in the results. The rest of the parameters, however, played an important role in the protocol efficiency (Supplementary Table 1). Here, only the results associated with changes in the phloroglucinol concentration will be presented in detail.

#### 3.1.1 Effect of changes in phloroglucinol concentration

The phloroglucinol (PG) is a key component of the colorimetric assay since it reacts with the furfural molecules forming a pink-red phloroglucide precipitate whose intensity is associated with the content of the pentosans in the analyzed sample (Kiszonas et al., 2012). For this reason, changes in the concentration of phloroglucinol in the extraction solution could affect the results. In order to determine whether the phloroglucinol concentration is a limiting factor and to identify the best phloroglucinol concentration to be used to both reliably estimate the pentosan concentration in a sample and to optimize the use of this chemical, four control lines with relatively high and low TOT-AX concentration were analyzed using an extraction solution that had a phloroglucinol concentration varying from 75% less to 75% more phloroglucinol concentrations compared to the solution reported by Hernández-Espinosa et al. (2020). As reported in the Supplementary Table 2 as the amount of PG decreases, the concentration of AX detected increased and vice versa. For instance, for the TOT-AX fraction, the greatest decrease in the concentration of AX in refined flour and whole meal occurs when using 75% more PG, whereas for the WE-AX the maximum decrease occurs at 50% more PG in refined flour and 25% more of PG in whole meal flour. The opposite effect (higher concentrations) occurs using 25% less PG concentration for the TOT-AX fraction in refined flour and 75% less PG in whole meal flour. For the soluble fraction, decreasing 25% of PG results in the highest concentrations in both types of flour (Supplementary Table 2).

Since the publication of the modified phloroglucinol colorimetric assay by Douglas (1981), few studies have investigated the effect of PG variation on AX concentration in a wheat flour sample. Kiszonas et al. (2012) reported that by using a PG concentration of 10% or 20% (original PG concentration used by Douglas (1981) and Hernández-Espinosa et al. (2020)), no significant differences could be observed in the absorbances obtained. In the same study, an increase in 40% of the PG compared to the control was associated with markedly lower absorbance values likely due to the excessive amount of phloroglucinol for the quantity of pentosans present in the sample. These results are comparable to those obtained in the present study. The AX quantification method proposed in this study uses 20% PG for the quantification of the TOT-AX in whole meal (originally proposed by Douglas (1981) and 10% PG for the quantification of the TOT-AX and WE-AX fraction in refined flours, and for the quantification of the WE-AX fraction in whole meal flour. It has been shown (Supplementary Table 2) that these PG concentrations are sufficient to perform a correct detection of furfural compounds comparable to the one obtained using the 20% PG concentration and avoid overestimating or underestimating the AX concentrations. Reducing the PG concentration also lowers the overall cost of the assay, thus improving its cost-effectiveness.

#### 3.1.2 Protocols comparisons and validation

After optimizing the different parameters that could influence the protocol performance, the PentoQuant final protocol was defined (Supplementary Fig. 1). The new method was validated with 16 spring bread wheat samples which were also analyzed using gas chromatography, the phloroglucinol colorimetric assay reported by Finnie et al. (2006), the phloroglucinol assay reported by Hernández-Espinosa et al. (2020), and the updated phloroglucinol colorimetric assay reported in the present study. Gas chromatography was used to measure only the TOT-AX fraction from RF and WM; the colorimetric methods reported by Finnie et al. (2006) and Hernández-Espinosa et al. (2020) were used to analyze both the TOT and WE-AX fractions in RF, and the PentoQuant was used to analyze both the TOT-AX and WE-AX fraction in RF and WM (Supplementary Table 3).

**Fig. 1:**
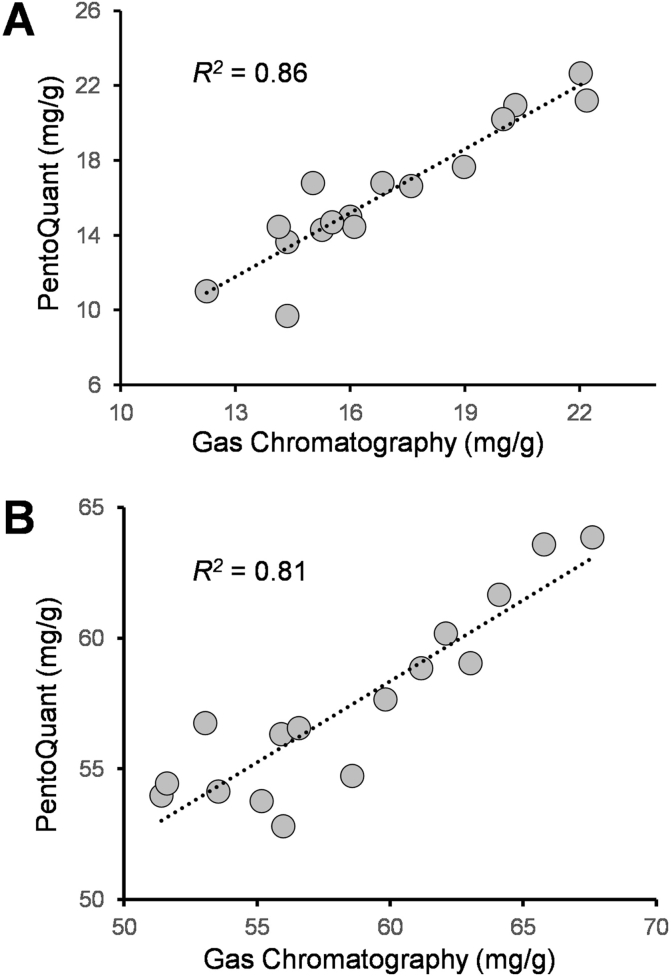
Comparison of the total arabinoxylan (TOT-AX) content obtained in (A) refined flour and (B) whole meal using the PentoQuant method and gas chromatography.

When comparing the four different methods used in this study through an ANOVA analysis (Table 1), in most cases it appeared that the method of analysis had a significant influence on the obtained AX concentration. Least-square mean comparison analysis showed that for the TOT-AX in WM or RF, no significant differences could be observed between the results obtained through chromatography and the ones obtained with the method proposed in this study. The results obtained by using the method proposed by Finnie et al. (2006), or Hernández-Espinosa et al. (2020) were comparable with each other but significantly different from the results obtained through either chromatography or the new colorimetric method (Table 1). These results were confirmed also through the Pearson's correlation analysis which indicated the presence of a strong positive correlation between the TOT-AX values obtained through chromatography and those obtained with the PentoQuant method (*r* = 0.83–0.93) (Fig. 1). On the contrary, the correlation between the RF TOT-AX values obtained through the protocol from Finnie et al. (2006) and those obtained through either chromatography or the new colorimetric method, were the lowest (*r* = 0.16 and 0.31 respectively). Differences in the protocol performance and the absence of the sulfuric acid solubilization step in the protocol reported by Finnie et al. (2006) could partly explain the observed variations. Overall, the TOT-AX results obtained with the new colorimetric method appeared to be the most correlated to those obtained through gas chromatography in both types of flour suggesting that the colorimetric method proposed in the present study would be the best among the three colorimetric assays used to quantify the TOT-AX content.

**Table 1:** Effect of the different extraction and quantification methods, on the total (TOT-AX) and water-extractable (WE-AX) arabinoxylan concentration detected in a set of 16 different whole meal and refined flour samples.

AX Fraction	Flour	AX Analysis Method	Lsmeans (mg/g)^a^	ANOVA	
				F-Value	*R*^*2*^
TOT-AX	Whole Meal	Gas chromatography	58.5	ns	0.02
		PentoQuant	57.4		
TOT-AX	Refined Flour	Gas chromatography	16.9a	4.38**	0.11
		Finnie et al. (2006)	14.0b		
		Hernández-Espinosa et al. (2020)	14.4b		
		PentoQuant	16.3a		
WE-AX	Refined Flour	Finnie et al. (2006)	4.3b	9.99***	0.18
		Hernández-Espinosa et al. (2020)	6.3a		
		PentoQuant	5.8a		

**, *** significant at *p* ≤ 0.01 and 0.0001, respectively. ns = non-significant.

a Values associated with different letters are significantly different at *p* ≤ 0.05.

Regarding the WE-AX fraction in refined flour, only the three colorimetric assays from Finnie et al. (2006); Hernández-Espinosa et al. (2020) and the PentoQuant, were compared. Significant differences in the WE-AX concentration were observed and, in this case, only the results obtained following the protocol of Finnie et al. (2006) were significantly different from the rest of the results (Table 1). Indeed, the results obtained using the method from Hernández-Espinosa et al. (2020) and the new colorimetric method were highly correlated (*r* = 0.94) whereas the method from Finnie et al. (2006) was the one that was the least correlated with the results obtained from the other two colorimetric methods (*r* = 0.71).

All the three tested colorimetric methods were conducted in duplicate and when comparing the average (AVG) standard deviation (StDev) and coefficient of variations (CV) associated with the analysis of the TOT-AX and WE-AX fractions in refined flour, the PentoQuant was associated with the lowest values (Supplementary Table 4). Specifically, for the TOT-AX values, the results obtained with the method reported in this study were associated with an AVG StDev and CV values of 1.11 and 6.50, respectively, whereas the method of Finnie et al. (2006) was associated with AVG StDev of 3.08 and AVG CV of 22.45 and the method of Hernández-Espinosa et al. (2020) was associated with AVG StDev of 1.99 and AVG CV of 14.66. Similarly, the WE-AX values obtained in RF using the PentoQuant, were associated with the lowest AVG StDev (0.39) and CV (6.90) values, compared to the other two colorimetric methods (Finnie et al., 2006: AVG StDev = 0.99, AVG CV = 21.84; Hernández-Espinosa et al., 2020: AVG StDev = 1.03, AVG CV = 17.29) (Supplementary Tables 3 and 4). Based on these results, the PentoQuant colorimetric protocol showed to be more repeatable compared to the other two analyzed colorimetric methods suggesting that it could be used as a reliable tool for arabinoxylan quantification in wheat flour.

### 3.2 Total and water extractable AX content variability

**Fig. 2:**
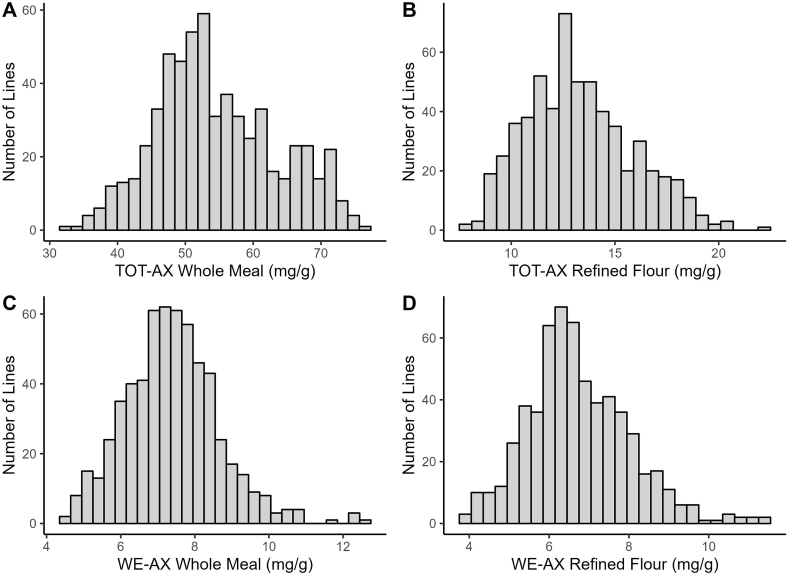
Distribution of the total arabinoxylan (TOT-AX) content obtained in (A) whole meal and (B) refined flour and of the water extractable arabinoxylan (WE-AX) content obtained in (C) whole meal and (D) refined flour. The results were obtained from the analysis of 606 spring common wheat lines analyzed in one environment.

The newly developed method for the AX quantification was tested on 606 whole meal and refined flour samples obtained from unique spring bread wheat lines developed by the CIMMYT bread wheat breeding program. To the best of our knowledge, this is the largest study conducted on wheat, for the characterization of the AX flour concentration. According to the obtained results, wide and continuous variation was identified in both the TOT and WE-AX fraction in either whole meal or refined flour (Fig. 2). Specifically, the observed TOT-AX concentration ranged from 31.9 to 76.1 mg/g in WM and 7.7–22.4 mg/g for RF (Supplementary Table 5). As expected, an average 74% reduction in the TOT-AX content could be observed in the refined flour, which is associated with the removal of the aleurone and pericarp layers (Saulnier et al., 2007). Comparing these results with other previously published studies, the variation of TOT-AX in RF was higher than the one reported by Hernández-Espinosa et al. (2020) (10.8–16.5 mg/g) and Finnie et al. (2006) (7.9–19.1 mg/g) but comparable to the one reported by Gebruers et al. (2008) (13.5–27.5 mg/g). Also, the variation of the TOT-AX WM content was slightly higher compared to the variation identified in previously published report (Dornez et al., 2008, 58.1–70.9 mg/g; Finnie et al., 2006, 30.6–63.6 mg/g) but still the values obtained were comparable. The differences of TOT-AX in whole meal and refined flour across studies were likely determined by the different genotypes analyzed and by the environments the wheat lines were grown. Additional changes could be determined by the laboratory protocol, especially by the utilization or not of the sulfuric acid solubilization step for the TOT-AX quantification. Among the analyzed germplasm, the two lines GID8240480 and GID8238785 exhibited a high concentration for total fraction in both types of flour (74.2 mg/g in WM and 20.3 mg/g in RF; 71.2 mg/g for WM and 22.4 mg/g in RF, respectively). Even though these results must be confirmed by additional analysis, these two lines could represent good sources for high TOT-AX content in the CIMMYT breeding program (Supplementary Table 5).

Concerning the WE-AX fraction, the analyzed samples exhibited values ranging from 4.4 to 12.6 mg/g in WM and from 3.9 to 11.4 mg/g in RF. In this case, the lines GID8238593 and GID8238785 presented the highest WE-AX concentration in WM whereas the lines GID8241197 and GID 8239381 constituted a simultaneous good source of both AX fractions for refined wheat flour (Supplementary Table 5). The WE-AX content values obtained in this study were slightly higher than those reported by Finnie et al. (2006) (RF = 1.4–8.1 mg/g; WM = 2.4–9.1 mg/g), Török et al. (2019) (RF and WM = 1.3–7.6 mg/g) and Vignola et al. (2016) (RF = 3.5–7.2 mg/g; WM = 3.5–7.5 mg/g) but comparable to the WE-AX concentrations identified by Gebruers et al. (2008) in a set of 131 bread wheat winter lines (3.0–14.0 mg/g).

When comparing the content of the two fractions, significant and positive correlations were identified between the TOT-AX and WE-AX in WM (*r* = 0.18) and RF (*r* = 0.52). Higher correlation values could be observed for the TOT-AX contents identified in whole meal and refined flour (*r* = 0.71) and the WE-AX content identified in the refined and unrefined samples (*r* = 0.55). These results are in partial agreement with the results previously reported in the literature. Vignola et al. (2016) for example, did not find a significant correlation in the TOT and WE-AX content measured in whole meal and white flour. However, some other studies found higher and significant correlations between the TOT-AX and WE-AX fraction in refined flour, like Tremmel-Bede et al. (2020) and Hernández-Espinosa et al. (2020) who reported correlation values of 0.67 and 0.48, respectively. Similarly, Shewry et al. (2010) and Török et al. (2019) found correlations between the TOT-AX and WE-AX fraction of whole meal, of *r* = 0.30 and 0.37, respectively, and of *r* = 0.40 and 0.57, respectively, for refined flour.

Overall, these results indicate that samples with high TOT-AX content do not necessarily have high WE-AX content, and vice versa. Nevertheless, they also suggest that it is highly likely that the genotypes that exhibit high TOT-AX or WE-AX concentration in whole meal, also exhibit a high concentration of one of these two fractions in refined flour.

### 3.3 Association between molecular markers and AX content

The same 606 spring common wheat lines were also characterized with four KASP markers targeting the 1B QTL identified by Lovegrove et al. (2020) and Ibba et al. (2021), and with an additional marker targeting the 6B QTL identified by Mitchell et al. (2023) and associated with a peroxidase gene. The 1B QTL markers appeared to be significantly associated with variation in both the TOT and WE-AX content in either refined flour or whole meal (Table 2). The alleles associated with higher AX concentrations were present in around 25% of the lines, suggesting that these alleles are not rare and that they could be relatively easily selected for in elite germplasm. Similarly, to what reported by Ibba et al. (2021), the marker BA00789946 gave slightly different results compared to the markers 1B_653086336 and 1B_653681771, suggesting that the marker reported by Lovegrove et al. (2020) and those reported by Ibba et al. (2021) are not perfectly linked. The two markers reported by Ibba et al. (2021) were associated with almost identical results confirming that they are genetically linked and that could be used interchangeably. In terms of percentage of variability explained, on average all the 1B markers explained a lower phenotypic variation (*R*^*2*^ = 0.005–0.197) compared to what reported by Ibba et al. (2021) for the same markers. These differences could be due to either the type of germplasm analyzed and its growing conditions, or to the different method used to measure AX content. Like in Ibba et al. (2021) however, the two markers 1B_653086336 and 1B_653681771 were highly associated with changes in AX content and especially with variations in the soluble AX fraction where they explained, respectively, 19.6% and 19.7% of the variation in refined flour, and 10.5% and 10.1% of the variation in whole meal flour (Table 2).

**Table 2:** Effect of the three 1B specific and one 6B specific markers on the variation of the total (TOT-AX) and water-extractable arabinoxylan (WE-AX) content. Effects are expressed as *R*^*2*^ from the ANOVA analysis.

Marker	Whole Meal				Refined Flour			
	TOT-AX		WE-AX		TOT-AX		WE-AX	
	F-value	*R*^*2*^	F-value	*R*^*2*^	F-value	*R*^*2*^	F-value	*R*^*2*^
BA00789946	3.75*	0.01	18.87***	0.06	5.61**	0.02	32.08***	0.11
1B_653086336	6.23**	0.02	32.21***	0.11	12.01***	0.04	66.60***	0.20
1B_653681771	5.81**	0.02	30.66***	0.10	10.92***	0.04	66.74***	0.20
AX_94816599	ns	0.01	13.52***	0.05	ns	0.01	8.68***	0.03
1B_653681771	ns	0.03	4.26*	0.15	ns	0.05	19.55***	0.23
AX_94816599	ns		3.65*		ns		8.10***	
1B_653681771*AX_94816599	ns		ns		ns		ns	

*, **, *** significant at *p* ≤ 0.05, 0.01 and 0.001, respectively. ns = non-significant.

The 6B QTL marker was significantly and non-significantly associated with changes in the TOT-AX content in WM and RF, respectively. Interestingly, even if with a lower effect compared to the markers specific for the 1B QTL, variations of this marker were highly significantly associated with changes in the WE-AX fraction in both types of flour (Table 2). These results are in agreement with those reported by Mitchell et al. (2023) where it was reported that this marker is associated with a single missense mutation at a peroxidase gene which was found to be associated with decreased cross-linking of the WE-AX chains and increased solubility of AX. The allele associated with higher WE-AX concentrations was present in only 43 lines (7.1%) thus less frequent than the 1B QTL (Supplementary Table 5). Selection of this allele is desirable to additionally improve the WE-AX content in wheat.

**Fig. 3:**
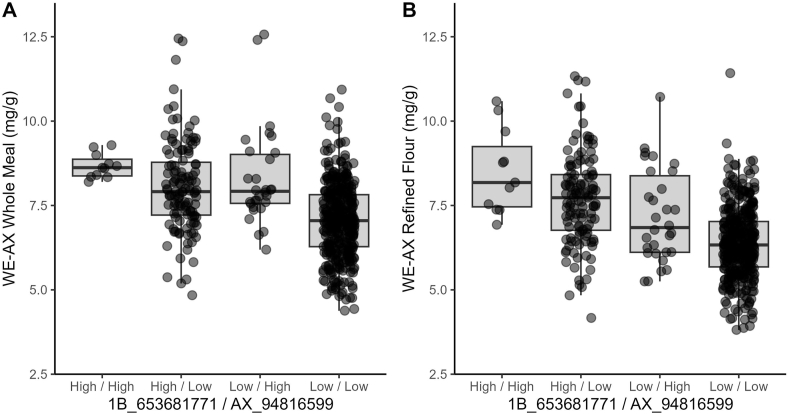
Distribution of water-extractable arabinoxylan (WE-AX) content in (A) whole meal and (B) refined flour samples, in relation to the combination of alleles identified using the 1B-specific KASP marker 1B_653681771 and the 6B-specific KASP marker AX_94816599.

To evaluate the combined effect and possible interactions of the 1B and 6B QTL, an ANOVA was also conducted including the 1B QTL marker 1B_653681771 and the 6B QTL marker. No significant interaction between the 1B and the 6B markers was identified. Nevertheless, the model combining the two markers was able to explain a greater amount of variation compared to the models including only the single markers and, especially for the WE-AX fraction, both the 1B and 6B markers appeared to have a significant effect on the observed phenotype suggesting that their effect is additive (Table 2) (Fig. 3).

Based on the results obtained, the 1B and 6B QTL specific markers previously published, could be effectively used, particularly in the identification of wheat lines with higher than average WE-AX content. Nevertheless, the discovery of additional alleles associated with AX content and more specifically tailored to the TOT-AX fraction, is essential to significantly improve the overall dietary fiber content of wheat.

## 4 Conclusions

The widely employed AX colorimetric quantification method, originally proposed by Douglas (1981), has been successfully modified to enhance repeatability, throughput and sample handling compared to other commonly used methods for AX quantification. This modified protocol, here named PentoQuant, was employed to measure the TOT-AX and WE-AX variability in a collection of 606 spring common wheat lines derived from the CIMMYT breeding program revealing the presence of a wide variation in the arabinoxylan concentration. Moreover, the analysis of AX content variation in these lines confirmed the associations between the observed AX variability and four distinct molecular markers targeting two major AX QTL on the 1BL and 6BS chromosomes. The combined utilization of these molecular markers and the PentoQuant method constitutes a powerful tool to assist in the selection of wheat lines with higher AX content.

## CRediT authorship contribution statement

**Nayelli Hernández-Espinosa:** Writing – review & editing, Writing – original draft, Methodology, Formal analysis, Data curation, Conceptualization. **Gabriel Posadas-Romano:** Writing – review & editing, Methodology. **Susanne Dreisigacker:** Writing – review & editing, Methodology, Data curation. **Jose Crossa:** Writing – review & editing, Methodology. **Leonardo Crespo:** Resources. **Maria Itria Ibba:** Writing – review & editing, Visualization, Supervision, Resources, Conceptualization.

## Declaration of competing interest

The authors declare that they have no known competing financial interests or personal relationships that could have appeared to influence the work reported in this paper.

## Data Availability

Data will be made available on request. Replication Data for: Efficient Arabinoxylan Assay for Wheat: Exploring Variability and Molecular Marker Associations in Wholemeal and Refined Flour (Original data) (CIMMYT Research Data & Software Repository Network)
